# The effect of experimental warming on reproductive performance and parental care in the burying beetle *Nicrophorus nepalensis*

**DOI:** 10.1098/rsos.240653

**Published:** 2024-10-09

**Authors:** Tanzil Gaffar Malik, Benjamin J. M. Jarrett, Syuan-Jyun Sun

**Affiliations:** ^1^ International Degree Program in Climate Change and Sustainable Development, National Taiwan University, Taipei 10617, Taiwan; ^2^ School of Environmental & Natural Sciences, Bangor University, Bangor, Gwynedd LL57 2UR, UK

**Keywords:** climate change, reproductive success, life-history traits, nest construction

## Abstract

Rising temperatures can adversely affect parental care and reproductive performance across a range of taxa. However, the warming impact is contingent upon understanding how temperature affects the spectrum of parental behaviours and their interplay. Here, we assessed how temperature affects parental care and reproductive success in the burying beetle, *Nicrophorus nepalensis*, which exhibits complex parental care behaviours. We exposed breeding pairs of *N. nepalensis*, to three temperature regimes (18°C, 20°C and 22°C) and assessed changes in parental care, and the subsequent development and growth of their offspring. Our findings show that 22°C disrupts carcass nest building by the parents and results in smaller clutches. Moreover, no eggs successfully hatched in the 22°C treatment. A milder increase to 20°C did not affect the hatching rate but resulted in smaller broods and lighter offspring, even when considering brood size, suggesting a change in post-hatching care quality. Our research suggests that warming may weakly affect parental care but has strong detrimental effects on offspring performance. These findings highlight the necessity of investigating the effect of ambient temperature across a diversity of traits and behaviours and across a range of life-history stages to fully assess species vulnerability in the face of future climate change.

## 1. Introduction

Anthropogenic climate change is exerting a profound influence on global biodiversity, reshaping ecosystems and pushing numerous species to extinction [1,2]. This escalating crisis stems from the dual challenges species face: the degradation of critical habitats and resources due to climatic shifts and species’ limited capacity to adapt to these rapid environmental changes in time [3]. Nevertheless, some species demonstrate resilience through various mechanisms, including both evolutionary adaptations and behavioural plasticity, allowing them to withstand the effects of climate change [4]. Behavioural plasticity involves immediate and flexible responses to environmental changes without genetic alterations [5], and as such, offers a rapid and effective means for species to mitigate the detrimental impacts of harsh environmental conditions, like increasing temperature. This is especially crucial for ectothermic organisms, particularly arthropods [6,7], which demonstrate heightened sensitivity to thermal fluctuations [8–10]. Understanding these behavioural adjustments is crucial for assessing a species’ vulnerability and potential for survival, as thermal conditions are critical determinants of numerous traits related to fitness such as mating behaviours, parental investment, locomotion and foraging efficacy [11–13] (but see [14]). Not all of these traits may be affected by temperature to the same extent or even in the same direction, underscoring the need to comprehensively understand how multiple traits respond to a warming world.

The reproductive ecology of burying beetles (Staphylinidae: *Nicrophorus* spp.) offers an intricate example of a taxon with a suite of behavioural adaptations that may be altered in response to a change in environmental conditions: elaborate parental care. Reproduction in burying beetles depends solely on the availability of small vertebrate carcasses such as mammals and birds. Once the beetles locate a carcass, they remove any fur or feathers, spread it with antimicrobial secretions before burying it underground as an edible nest [15,16]. This anal exudate-laying behaviour can effectively reduce bacterial growth during breeding (i.e. both before and after hatching of larvae [17]). Direct manipulation of carcass into the nest ceases prior to larval hatching when the carcass is ensconced in a burial chamber [18]. As the larvae hatch and develop, the parents provide elaborate parental care by directly offering regurgitated food to their larvae [18–20]. Parental care in *Nicrophorus* therefore forms a suite of stage-dependent complex parental behaviours that involves pre-hatching care (e.g. cooperation between both parents to prepare the carcass nest [21–23]) and post-hatching care (e.g. offspring provisioning and carcass maintenance [18]). Extreme temperatures can disrupt the processes of parental care and the development of offspring. In *N. vespilloides*, higher temperatures were detrimental to the offspring, and the evidence was mixed regarding whether parental presence could effectively mitigate the thermal stress [24]. Similarly, empirical evidence from *N. orbicollis* showed that high ambient temperatures impeded reproductive initiation and resulted in smaller broods [25], though this decrease in brood size may be attributable to a strategic reallocation of parental resources, wherein *N. orbicollis* parents intentionally limit brood size to increase care per larva [26]. Such a lack of consensus could be clarified by a more detailed investigation on the impact of ambient temperature on the diversity of behaviours that compose parental care. The overarching implications of these findings underscore the importance of reproductive behaviours and their outcomes as a function of thermal variation. To forecast the effects of global warming on reproductive behaviours and fecundity with precision, it is imperative to assess the thermal resilience of these species’ behavioural traits and across a range of life-history stages.

Here, we investigate how elevated temperatures affect reproductive behaviour and offspring performance in the burying beetles *N. nepalensis*, which provide biparental care to their offspring. We assessed a spectrum of reproductive traits including reproductive investment (clutch size), and proxies for pre-hatching care (i.e. carcass preparation) and post-hatching care, by investigating offspring development metrics such as brood size, total brood mass and average larval mass across these temperature treatments. Given the pivotal role of reproductive behaviours in determining fitness outcomes and their potential susceptibility to temperature fluctuations, we hypothesize that warmer temperatures will negatively impact the parental care provided by parents. Consequently, we predict that beetles reproducing at higher temperatures will perform carcass preparation less effectively, have fewer offspring and produce lighter offspring.

## 2. Material and methods

### 2.1. Study system and colony maintenance

All burying beetles were descendants of a stock population that was established in 2022 from beetles collected from six different sites near Shenkeng District in northern Taiwan (24.99° N, 121.62° E). No permissions were required prior to conducting this field collection. All beetles were checked and mites were removed if present, before beetles were introduced to laboratory colonies. Beetles were housed individually in plastic containers (10.8 × 7.5 × 2.1 cm) filled with moist soil and fed with minced pork (approx. 1.5 g) twice a week before reaching sexual maturity. We reared the beetles in standardized laboratory conditions on a 10 : 14 light-to-dark cycle, 70% relative humidity and the temperature was adjusted to replicate the daily temperature fluctuation (average 17.8°C, range of fluctuations 16°C–20°C), which is typical of the beetle’s breeding season from November to April in northern Taiwan [27]. Previous work also showed that natural populations of *N. nepalensis* were most abundant in temperatures ranging from 15°C to 20°C, and the temperature for optimal performance in reproduction peaked around 16°C [27].

### 2.2. Experimental temperature treatments

To investigate the effect of temperature on the reproductive behaviours and performance of *N. nepalensis*, we used three sets of beetles, each subjected to a distinct range of temperatures: control (*n* = 28), an increase of 2°C (*n* = 25) and an increase of 4°C (*n* = 28), in accordance with the projected global warming scenarios [28]. An increase of 2°C and 4°C represent a moderate and extreme temperature increase, respectively, relative to 2011–2020, which was already more than 1°C warmer than the pre-industrial levels [28].

The temperatures were maintained under diurnal temperature fluctuations using programmable incubators (Hipoint, FH-740). The ambient temperature (control) was set to vary between 16.8°C and 20.1°C, and the moderate warming condition between 18.8°C and 22.1°C, and the heightened warming condition between 20.8°C and 24.1°C. We bred pairs of unrelated beetles that were haphazardly selected from the stock population and provided 20–30 g (25.78 ± 2.30; mean ± s.d.) newly thawed mouse carcasses in breeding boxes (14.2 × 6.3 cm) filled with moist soil (to a depth of 2 cm). Note that this depth of soil could inhibit beetles from burying carcasses completely. We acknowledge that the soil depth in the lab will almost always be limited and may not offer the same properties of a thermal barrier and heat sink as it may do in nature, which may allow pairs to buffer temperature effects. However, the beetles were still able to partially bury carcasses at this depth, which allowed us to accurately observe carcass nest building and record time until the first larva hatched.

These boxes were then placed in the incubator on the darkened shelf to mimic the natural breeding environment underground. Since pairing, we monitored each pair for the presence of eggs by looking at the bottom of the breeding box at an interval of 8 h. This estimation of clutch size allowed us to accurately predict the actual clutch size (Pearson’s correlation *r* = 0.898, *p* < 0.001, *n* = 77 broods). We also monitored carcass preparation and presence of first-instar larva. Carcass preparation was determined as successful when the fur was completely removed from the carcass, and it had been rounded into a ball. Unsuccessful carcasses had any fur still attached and were not rolled into a ball. Note that male abandonment during breeding is common across *Nicrophorus* spp. [29,30]. In our study, we observed a few males abandoning the breeding carcass, leaving only the females to provide further parental care. This could potentially cause some variation in the reproductive output we recorded. However, all males were kept within the boxes until larval dispersal, so this male abandonment should have had limited effects. After larval hatching, all broods were checked daily for larval dispersal, i.e. at least one third-instar larva was found leaving the carcass to pupate in the soil nearby. At this point, we collected and counted all third-instar larvae, and weighed the whole brood to the nearest 0.001 g (Shimadzu; Model: ATX224R). We also determined the averaged larval mass (brood mass divided by brood size), as this is commonly used as a measure of larval body size. Higher larval mass results in larger adult body size, which confers fitness advantage in terms of reproduction [31] and fighting ability [32].

### 2.3. Statistical analyses

All analyses were conducted in R (v. 4.1.2 [33]), using generalized linear mixed models (GLMM) with the *glmer* function in the ‘lme4’ package [34]. In all analyses, temperature treatment (18°C, 20°C and 22°C) was included as a categorical explanatory variable, and carcass mass was included as a covariate. Male and female families were included as random effects since 1–7 individuals from 43 different families were used (18°C: 1–4 individuals from 34 families; 20°C: 1–5 individuals from 30 families; 22°C: 1–4 individuals from 34 families).

### 2.4. Effect of temperatures on carcass preparation

We analysed the effect of the temperature treatment on carcass preparation by the parents, with proportion of carcasses prepared and time until carcass preparation as dependent variables. The proportion of carcasses prepared was analysed using a binomial distribution using a cloglog link to correct for the imbalance of 0 and 1 in the dataset, whereas time until carcass preparation was analysed using a Gaussian distribution.

### 2.5. Effect of temperatures on reproductive investment

We analysed the effect of the temperature treatment on reproductive investment, using egg laying time and clutch size. Egg laying time was log-transformed prior to analysis to meet the normality assumptions of model residuals for a Gaussian distribution, whereas clutch size was analysed using a Poisson distribution.

### 2.6. Effect of temperatures on offspring development and growth

We analysed the effect of the temperature treatment on the time to the presence of first larva, the time to larval dispersal, as well as brood size and brood mass. For the analysis of brood size and brood mass, we only considered broods with eggs that hatched. The time to the presence of first larva, the time to larval dispersal and brood mass were log-transformed prior to analysis using a Gaussian distribution, whereas the brood size was analysed using a Poisson distribution. Furthermore, to test whether the temperature treatment also influenced averaged larval mass, we included the temperature treatment and brood size as a covariate.

## 3. Results

**Figure 1.**
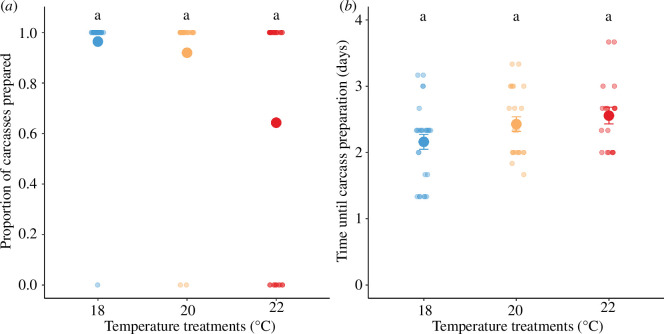
The effect of temperature treatments on parental behaviour, quantified from (*a*) the proportion of carcasses prepared and (*b*) the time until carcass preparation. Individual data were pointed with a jitter effect to prevent overlap. The letters denote non-significant differences among temperature treatments (Tukey honestly significant difference (HSD) tests).

The proportion of carcasses fully prepared decreased with temperature (18°C = 96%, 20°C = 92% and 22°C = 64%), though there was no statistical support for this effect (*χ*^2^ = 3.89, d.f. = 2, *p* = 0.143; figure 1*a*). Among the pairs that successfully prepared the carcass into a ball, there was a tendency that beetles in warming environments spent more time until carcass was fully prepared (*χ*^2^ = 4.96, d.f. = 2, *p* = 0.084; figure 1*b*).

**Figure 2.**
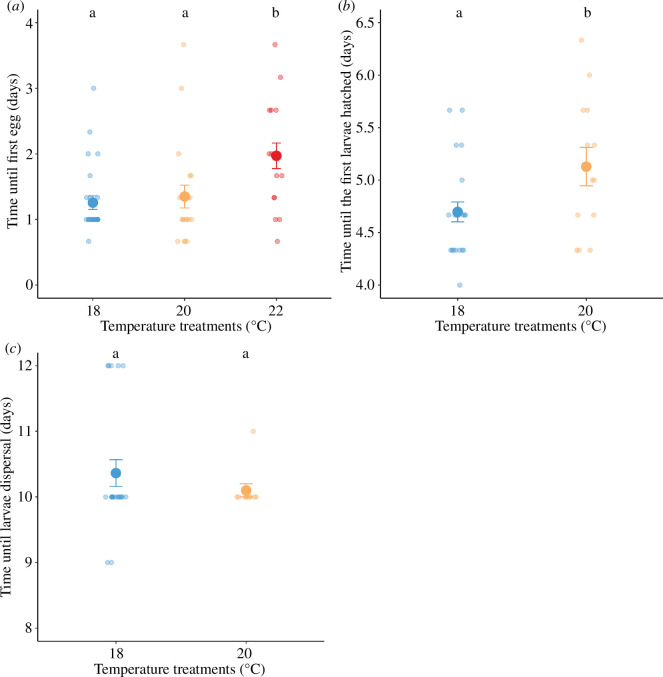
The effect of temperature treatments on the development and growth of larvae, as evidenced from (*a*) the number of days until the first egg is laid, (*b*) the number of days until the first larva hatched and (*c*) the number of days until larval dispersal. Individual data were pointed with a jitter effect to prevent overlap. Different letters denote significant differences among temperature treatments (Tukey HSD tests).

**Figure 3.**
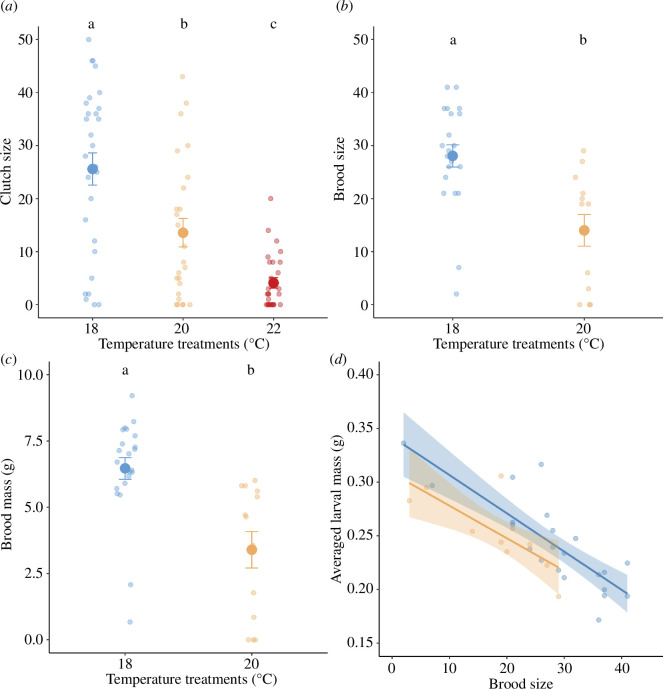
The effect of temperature treatments on the reproductive performance measured as (*a*) clutch size, (*b*) brood size and (*c*) brood mass, whereas (*d*) the averaged larval mass negatively correlated with brood size. Individual data were pointed with a jitter effect to prevent overlap. Lines are statistically significant relationships predicted from GLMMs, whereas shaded areas represent 95% confidence intervals. Different letters denote significant differences among temperature treatments (Tukey HSD tests).

Experimental warming also affected the reproductive investment of burying beetles, delaying the time until first egg laid (*χ*^2^ = 19.28, d.f. = 2, *p *< 0.001; figure 2*a*). Specifically, 22°C but not 20°C led to prolonged oviposition compared with 18°C (post hoc comparison 18°C versus 22°C, estimate = −0.33, s.e. = 0.08, *t* = −3.88, *p *< 0.001; 18°C versus 20°C, estimate = −0.04, s.e. = 0.08, *t* = −0.49, *p* = 0.877). Furthermore, beetles laid significantly fewer eggs at higher temperatures (*χ*^2^ = 58.69, d.f. = 2, *p *< 0.001; figure 3*a*). Of those broods with eggs, none of the eggs hatched at 22°C. Hatching rate was similar between 18°C and 20°C treatments (*χ*^2^ = 2.00, d.f. = 1, *p* = 0.157). Warming to 20°C delayed the time until the first larva hatched (*χ*^2^ = 6.09, d.f. = 1, *p* = 0.014; figure 2*b*) but had no effect on the time until larval dispersal (*χ*^2^ = 0.81, d.f. = 1, *p* = 0.368; figure 2*c*) relative to 18°C.

Beetles exposed to warming to 20°C produced significantly smaller and lighter broods compared with the 18°C treatment (brood size: *χ*^2^ = 16.57, d.f. = 1, *p *< 0.001; brood mass: *χ*^2^ = 12.24, d.f. = 1, *p *< 0.001; figures 3*b,c*). Furthermore, individual larvae reared under +2°C also became smaller (*χ*^2^ = 5.44, d.f. = 1, *p* = 0.020; figure 3*d*) while controlling for the brood size, which negatively predicted lighter individual larval mass (*χ*^2^ = 49.95, d.f. = 1, *p *< 0.001; figure 3*d*).

## 4. Discussion

We found that the experimental warming conditions negatively influenced the reproductive performance of *N. nepalensis*. Our results show that increasing ambient temperature had minimal impacts on pre-hatching parental care, while an increase to 20°C, although moderate, reduced the reproductive investment of parents, with smaller clutches and broods, and lighter offspring. These results are consistent with other previous studies on burying beetles which showed that insect behaviour is highly sensitive to environmental factors, like temperature, that are currently undergoing rapid change across the world [35,36].

Ambient temperature can affect different behaviours and life-history traits across a range of developmental stages [37]. Our study found that increased temperatures had minimal impacts on the pre-hatching care behaviour of carcass preparation (figure 1*a*), while slightly increased the time it took for parents to complete carcass preparation (figure 1*b*), a result consistent with previous studies [35]. In other nest building species like birds, parents alter the characteristics of the nest in response to increased temperature. Nests of the zebra finch (*Taeniopygia guttata*) are lighter in warmer environments [38] and comprised different ratios of construction materials compared with nests built at cooler temperatures [38,39]. Adjustments of nest properties can act as a mechanism to buffer developing offspring from increased temperatures. In burying beetles, such a mechanism is not in place as their nest is a vertebrate carcass. Higher temperatures increase the rate of decomposition of the carcass, which effectively increases competition with microbes. Parent beetles may have been investing more heavily in defending the carcass from microbial competitors, which would explain why carcasses took longer to be stripped of fur and rolled into a ball. Future work should consider assaying expression of lyzozyme genes expressed by parents in warmer environments as an adaptive response to the greater microbial load on the carcass [38]. Our experimental set-up did not allow the carcass to be fully buried, which may be one behaviour that parents alter in warmer environments to buffer the carcass and is thus worthy of consideration. Investigating such an effect may be difficult in the laboratory as even a large amount of soil will not offer the same heat sink properties that earth in nature may provide.

While we did not measure post-hatching parental care directly, we found changes in average larval mass that are indicative of a reduction of post-hatching care. Specifically, average larval mass was lower in the 20°C treatment (figure 3*d*), even when taking brood size into account, which is consistent with previous studies on burying beetles [24,25]. An alternative hypothesis is that the carcass quality is lower at higher temperatures, and this impacted larval mass over and above the effect of the ambient temperature alone. Future research should decompose the effect of temperature on the multiple components of parental care to truly understand whether parental care is negatively affected by ambient temperature, or post-hatching parental care cannot overcome the negative effects of ambient temperature on pre-hatching care behaviours like carcass preparation. The multiple behaviours that compose parental care in burying beetles may not be equally affected by ambient temperature, and these behaviours may interact in complex, nonlinear fashion, complicating broad conclusions about the effect of temperature on parental care as a whole. For example, higher ambient temperatures are predicted to affect incubation time in birds as a mechanism to buffer embryos from lethal air temperatures [40], which will impact the frequency of foraging trips that parents can undertake. However, increased ambient temperatures have been shown to reduce offspring begging, presumably to reduce energy expenditure in warmer conditions [41]. Such effects may mask each other if not directly investigated, and disentangling effects of ambient temperatures on the different components of parental care is a promising avenue for future research.

At 22°C, we found that no eggs hatched. This is probably due to a failure of fertilization [42]. Fertility thermal limits are generally lower than the thermal limits at which organisms die and thus may be the limiting factor in determining the fate of a population in response to climate change, as a population that cannot successfully reproduce will face extinction [43,44]. When fertilization can occur, however, ambient temperatures can negatively impact fecundity too [44]. Increased temperatures reduce brood size across *Nicrophorus* species [24,25,35], which may be due to decreased investment in clutch size (as we have shown in figure 3*a*), larval survival during development or infanticide by parents [45].

The compound effects of temperature across all developmental stages, from egg to larvae to adult, will have dramatic demographic consequences for populations threatened by a changing climate. Understanding the vulnerability of a species to environmental changes requires a detailed evaluation of the interplay between ambient temperature and a diversity of life-history traits under projected climatic conditions. The findings of this study suggest that even a moderate increase in ambient temperature caused by climate change could pose a threat to the reproductive success and population persistence of *N. nepalensis*. Parents had fewer, smaller offspring (even accounting for brood size) at higher temperatures as a consequence of reduced parental investment in clutch size and modified pre- and post-hatching care behaviours. Thus, we anticipate a reduction in both population size and individual body size of burying beetles under future warming scenarios. This prospect raises urgent concerns regarding the long-term sustainability and ecological resilience of burying beetle populations and the critical ecosystem services they support within forest habitats amid global climate change. Moreover, these results have broader implications beyond burying beetles, extending to other species sensitive to temperature changes. Particularly, similar disruptions in parental care and reproductive success due to warming have been observed in birds and mammals [40,41,46]. Therefore, our findings underscore the widespread impact of climate change, highlighting the need for comprehensive studies on a diverse array of species to understand the broader ecological consequences of global warming.

## Data Availability

Data and relevant code for this research work are stored in GitHub: https://github.com/syuanjyunsun/warming_exp and have been archived within the Zenodo repository [47].
